# Uso de las modalidades diagnósticas pertenecientes a la imagenología dentofacial en la odontología forense. Revisión de la literatura

**DOI:** 10.21142/2523-2754-0904-2021-088

**Published:** 2021-12-09

**Authors:** José Alberto Castillo Páez, Liliber del Carmen Fajardo de Pérez, Angelo Giovani Moffa Barros

**Affiliations:** 1 Departamento de Estomatoquirúrgica, Facultad de Odontología de la Universidad de Carabobo. Valencia, Venezuela. josecastillo031285@gmail.com, liliberf@hotmail.es Universidad de Carabobo Departamento de Estomatoquirúrgica Facultad de Odontología Universidad de Carabobo Valencia Venezuela josecastillo031285@gmail.com liliberf@hotmail.es; 2 Facultad de Odontología de la Universidad de Carabobo. Valencia, Venezuela. angelomoffa18@gmail.com Universidad de Carabobo Facultad de Odontología Universidad de Carabobo Valencia Venezuela angelomoffa18@gmail.com

**Keywords:** imagenología dentofacial, radiografía panorámica, radiografía lateral de cráneo, tomografía computarizada de haz cónico (TCHC), radiografía digital, odontología forense, dentofacial imaging, panoramic radiography, lateral cephalogram, cone beam computed tomography (CBCT), digital radiography, forensic dentistry

## Abstract

**Objetivo:**

Describir el uso de las modalidades diagnósticas pertenecientes a la imagenología dentofacial en la odontología forense.

**Materiales y métodos:**

Se realizó una búsqueda en la base de datos de PubMed, Google Académico y SciELO con las palabras clave “Dentofacial Imaging”, “Panoramic Radiography”, “Lateral Cephalogram”, “Cone Beam Computed Tomography (CBCT)”, “Digital Radiography”, y “Forensic Dentistry”. Se seleccionaron 48 artículos publicados en inglés, de fechas recientes, buscando información que describiera el uso de las modalidades diagnósticas pertenecientes a la imagenología dentofacial en la odontología forense.

**Resultados:**

Las modalidades diagnósticas de la imagenología dentofacial incluyen dentro de las más relevantes para la odontología forense la radiografía panorámica, la radiografía lateral de cráneo, la radiografía posterioanterior de cráneo y la tomografía computarizada de haz cónico.

**Conclusiones:**

Estas modalidades, junto a la ejecución de análisis morfométricos, permiten al odontólogo forense la identificación de un cadáver, la estimación de la edad, el sexo e incluso la reconstrucción facial forense con fines identificativos.

## Abstract (en)

**Objective:**

To describe the use of diagnostic modalities for dentofacial imaging in forensic dentistry.

**Materials and Methods:**

A scientific search was carried out in the PubMed, Google Academic, and SciELO databases with the keywords "Dentofacial Imaging", "Panoramic Radiography", "Lateral Cephalogram", "Cone Beam Computed Tomography (CBCT)", "Digital Radiography", and "Forensic Dentistry". Forty-eight recently dated articles published in English were selected, seeking information to describe the use of diagnostic modalities pertaining to dentofacial imaging in forensic dentistry.

**Results:**

The most relevant diagnostic modalities of dentofacial imaging for forensic dentistry include panoramic radiography, lateral skull radiography, posterior-anterior skull radiography and cone beam computed tomography.

**Conclusions:**

The modalities described, together with morphometric analyses, allow forensic dentists to identify a corpse, estimate age, sex, and even facilitate forensic facial reconstruction for identification purposes.

## INTRODUCCIÓN

La imagenología dentofacial es una disciplina que se encarga del estudio de las estructuras de tejidos duros y blandos pertenecientes a la región orofacial, así como del diagnóstico por imágenes de patologías pertenecientes a estos tejidos ^1^. Tradicionalmente, las modalidades utilizadas en odontología para el diagnóstico por imagen comprenden la práctica de radiografías periapicales, radiografías panorámicas, radiografías laterales de cráneo, y en la actualidad se ha empleado el uso de tomografías computarizadas para visualizar la articulación temporomandibular u otra estructura ^2^.

Ahora bien, en odontología forense, estos mismos métodos pueden ser utilizados para determinar la identidad de un cadáver, establecer su causa de muerte, evaluar evidencias observables en una imagen radiográfica, realizar algún examen requerido, evaluar lesiones y, en antropología forense, para estimar la edad, el sexo o la genética ^3^. De hecho, la imagenología forense permite la interpretación y el diagnóstico radiográfico que ayudará posteriormente a resolver problemas legales ^4^. Así, en la presente investigación se plantea como objetivo describir el uso de las modalidades diagnósticas pertenecientes a la imagenología dentofacial en la odontología forense.

## MATERIALES Y MÉTODOS

Se realizó una búsqueda en las bases de datos de PubMed, Google Académico y SciELO con las palabras “Dentofacial Imaging”, “Panoramic Radiography”, “Lateral Cephalogram”, “Cone Beam Computed Tomography (CBCT)”, “Digital Radiography”, “Forensic Dentistry”. Se consideraron además factores como que el texto del artículo esté completo, se encuentre en formato PDF y su fecha de publicación, que comprendió datas recientes previas al desarrollo de esta investigación. El criterio de selección considerado para los artículos seleccionados fue que guardaran relación directa con el objetivo de la investigación, es decir, describir el uso de las modalidades diagnósticas pertenecientes a la imagenología dentofacial en la odontología forense. Se obtuvieron, finalmente, 48 artículos que componen las referencias de la presente revisión. Esta estrategia se describe detalladamente en la Tabla 1.

**Tabla 1:** Fuentes de información utilizadas

Base de datos	Número de artículos encontrados	Número de artículos seleccionados
PubMed	125	40
Google Académico	15	2
SciELO	26	6
Total	166	48

## MODALIDADES DIAGNÓSTICAS PERTENECIENTES A LA IMAGENOLOGÍA DENTOFACIAL

Actualmente, existen técnicas radiográficas que incluyen la proyección radiográfica con ortopantograma, es decir, la radiografía panorámica y la lateral de cráneo, que muestran una visión bidimensional de las estructuras anatómicas orofaciales. Además, existe la proyección mediante la tomografía computarizada de haz cónico, que permite una visión tridimensional de estas estructuras y permite su observación, estudio y diagnóstico en caso de haber afecciones ^5^^,^^6^.

Clínicamente, estos métodos son muy utilizados en la práctica odontológica, ya que facilitan el diagnóstico de caries o patologías orales, así como sirven para planear tratamientos prequirúrgicos o preortodónticos ^7^.

Por el contrario, en las ciencias forenses, esencialmente la odontología forense, la imagen radiográfica es beneficiosa para el proceso de identificación. En el caso del reconocimiento de víctimas de desastres, por ejemplo, puede ayudar a identificar y asociar distintas partes de los cadáveres encontrados, principalmente del cráneo; asimismo, sirve como soporte de la documentación necesaria para la individualización e identificación ^8^.

## EL USO DE LA RADIOGRAFÍA PANORÁMICA EN EL ÁREA

Inicialmente, la radiografía panorámica brinda una visión bidimensional para examinar radiográficamente la mandíbula; además, es relevante para el estudio de las vértebras cervicales, el volumen óseo, la detección de caries dental y la enfermedad periodontal. Aunque tiene ventajas, como la corta exposición del paciente a los rayos X y las bajas dosis de radiación, presenta desventajas como la baja calidad de la imagen en comparación con las radiografías intraorales, es decir, se aprecian imágenes distorsionadas geométricamente, así como magnificaciones y elongaciones de estructuras anatómicas, superposición de vértebras y presencia de imágenes fantasma. La presencia de maloclusiones, además, puede reflejar en la proyección cierta deformidad considerable de todo el complejo maxilomandibular ^9^^,^^10^. Así, dentro de la odontología forense, esta proyección sirve para el estudio del ángulo goniaco mandibular, la altura de la rama de la mandíbula y el ancho del ángulo bigonial para estimar edad y sexo ^11^.

Estudios como el de Lundberg *et al*. ^12^ lo demuestran, ya que determinaron que era posible identificar pacientes edéntulos solo mediante la observación de las características anatómicas de la forma y el tamaño de la mandíbula. Ellos evaluaron 19 radiografías panorámicas *pre mortem* y las compararon con 19 radiografías panorámicas *post mortem* de los mismos sujetos, a quienes se les tomó la proyección con diferentes lapsos entre una y otra (entre 4 meses y 6 años). Además, incluyeron 10 radiografías extras para complicar la tarea de comparación entre las imágenes. Los resultados obtenidos fueron bastante buenos, con un 100% y un 96% de certeza de compatibilidad entre las imágenes *pre mortem* y las *post mortem*.

De la misma forma, Saloni *et al*. ^13^ buscaron determinar el género de 200 pacientes mediante la observación de una radiografía panorámica y el análisis morfométrico de la rama mandibular, y concluyeron que la mandíbula es un excelente indicador del dimorfismo sexual mediante el análisis de morfométrico de la rama en una radiografía panorámica, ya que permite proyectar su ancho, grosor y altura. Observaron que todos estos aspectos eran mayores en el sexo masculino y menores en el sexo femenino, y obtuvieron resultados de un 37% de las radiografías estudiadas pertenecientes al sexo masculino y un 63% de las radiografías estudiadas pertenecientes al sexo femenino.

Por último, puede agregarse el trabajo de Memorando *et al*. ^14^, quienes buscaron determinar la relación entre la edad dental y la edad cronológica mediante el desarrollo del tercer molar en pacientes filipinos de los grupos etarios entre los 3 y 23 años de la División de Odontología Pediátrica del Centro Médico para Niños Filipinos. En este estudio se analizó el crecimiento del tercer molar derecho usando el método de Demirjian modificado, en 384 radiografías panorámicas (215 de pacientes masculinos y 169 de pacientes femeninos). De esta investigación se concluyó que el desarrollo del tercer molar se inicia aproximadamente a los 9 años y que su desarrollo completo se alcanza, aproximadamente, a los 19 años. Esto demuestra una relación bastante estrecha entre la edad dental y la cronológica, así como la importancia del uso de la proyección panorámica en la estimación de la edad.

## EL USO DE LAS RADIOGRAFÍAS POSTEROANTERIOR Y LATERAL DE CRÁNEO

En otro orden de ideas, la radiografía posteroanterior de cráneo da una vista bidimensional del cráneo en ese sentido, relacionada con la dirección de los rayos X. Esta y la proyección lateral de cráneo son excelentes para la planificación de tratamientos ortodónticos, la evaluación de la anatomía de la articulación temporomandibular y la mandíbula, esencialmente el mentón ^15^^,^^16^.

Para la odontología forense, este tipo de proyección es de utilidad para la observación de los senos paranasales, especialmente el seno frontal, ya que, como muchas estructuras anatómicas, este es único para cada individuo, incluso cuando se habla de gemelos univitelinos ^17^.

En este marco, Shireen *et al*. ^18^ llevaron cabo una evaluación radiomorfométrica del seno frontal y su relación con la edad y el sexo en una población saudí. Evaluaron 400 radiografías de igual número de sujetos (200 de sexo masculino y 200 de sexo femenino), entre 14 y 70 años, a las cuales les realizaron un análisis morfométrico utilizando el programa Adobe Photoshop CS3 y obtuvieron resultados muy interesantes. Destacaron que existen diferencias morfológicas notables entre el seno frontal de los hombres y el de las mujeres; a saber, en el cráneo masculino existe un porcentaje mayor de ausencia unilateral y bilateral del seno frontal con relación al cráneo de sexo femenino, así como una asimetría considerable de esta estructura anatómica en los cráneos masculinos más que en los femeninos, lo que indica que el seno frontal en el cráneo masculino presenta mayores variaciones anatómicas comparado con el cráneo femenino, es decir, esta estructura anatómica es de gran utilidad para estimar caracteres como el sexo.

En concordancia, la radiografía lateral de cráneo muestra una visión bidimensional en sentido lateral de los huesos de la cara y el cráneo, y sirve para evaluar relaciones óseas, patrones de crecimiento, definición ósea y procesos alveolares. Este tipo de radiografía queda corto para estudiar estructuras craneales individualmente, debido a la superposición de las estructuras anatómicas faciales y craneales que, como es sabido, son pares ^19^^,^^20^.

En odontología forense, este tipo de proyección puede utilizarse, por ejemplo, para la reconstrucción de rasgos faciales utilizando la forma de la mandíbula, como lo hacen Omran *et al*. ^21^, quienes estudiaron 100 radiografías laterales de cráneo con análisis cefalométrico para evaluar los puntos gonion y pogonion, y su relación con la longitud de la mandíbula. Al obtener esta referencia, fue bastante fácil la reconstrucción de rasgos faciales a partir de este dato aportado por la radiografía.

Por su parte, Belaldavar *et al*. ^22^ destacaron el uso de esta modalidad diagnóstica en la determinación del sexo. Analizaron 304 proyecciones (155 de pacientes femeninos y 149 de pacientes masculinos) de grupos etarios entre 18 y 30 años, de sujetos indios, y midieron el ángulo goniaco usando el *software* Adobe Photoshop; sus resultados marcaron una diferencia notable de las medidas de este ángulo en las mandíbulas masculinas y femeninas. El promedio de medida fue de 122,7° para el sexo femenino y 121,1° para el sexo masculino. Lo que denota un uso bastante resaltante de esta modalidad diagnóstica en la estimación de sexo para el odontólogo forense.

Para estimar la edad, Utama *et al*. ^23^ evaluaron las vértebras cervicales observables en radiografías laterales de cráneo. También estudiaron la edad dental de los dientes visibles y la compararon con la edad cronológica; tomaron 100 proyecciones y midieron el índice de crecimiento coronal dental observable, el crecimiento de las vértebras, y los compararon con la edad cronológica de los sujetos, obteniendo resultados promedio de edades vertebrales de 13,97 años, edades dentales de 14,06 años y 13,97 años de edad cronológica. Esto significa que había diferencias de 0,094 ± 1,52 años entre una y otra, es decir, diferencias mínimas, por lo que concluyeron que una radiografía lateral de cráneo puede ser bastante útil para un odontólogo forense al estimar edad cronológica.

## EL USO DE LA TOMOGRAFÍA COMPUTARIZADA DE HAZ CÓNICO (TCHC)

En otro orden de ideas, la tomografía computarizada de haz cónico (CBCT, por sus siglas en inglés) es una modalidad diagnóstica que brinda imágenes bidimensionales y tridimensionales de alta resolución en diferentes planos. Es ideal para el estudio de estructuras grandes, como la mandíbula y el maxilar, y para estructuras pequeñas, como la anatomía interna del diente ^24^.

Actualmente, la TCHC es útil en la cirugía maxilofacial para verificar la trayectoria de nervios en caso de odontectomías o colocación de implantes dentales, y para localizar patologías de los senos paranasales. En endodoncia, sirve para ubicar raíces o conductos accesorios, así como fracturas radiculares verticales. En ortodoncia, contribuye a la planificación de cirugías ortognáticas y a evaluar con detalle la articulación temporomandibular. Allí radica su importancia clínica para el diagnóstico y la planificación de tratamientos odontológicos ^25^.

Por otra parte, en odontología forense, los usos dados a la TCHC son bastante variados. Pueden mencionarse algunos, como estudiar el dimorfismo sexual observando el foramen magnum. Esta estructura anatómica es una de las de mayor importancia en la base del cráneo, y se localiza en la parte inferior de la sutura sagital, rodeada de la porción escamosa del hueso temporal y los lados laterales del hueso occipital, en la parte más profunda y posterior de la fosa craneal, y cubierta de un gran volumen de tejido blando. Ha demostrado tener una diferencia notable en su volumen según pertenezca a personas de sexo femenino o masculino ^26^.

Estudios como el de Mustafi *et al*. ^27^ respaldan lo mencionado, ya que ellos observaron 120 CBCT de 60 hombres y 60 mujeres a quienes se les midió los diámetros transversal y sagital, así como también la circunferencia del *foramen magnum*. Obtuvieron diferencias notables entre los diámetros transversales y las circunferencias de la estructura anatómica entre hombre y mujeres, mientras que, con relación al diámetro sagital, no encontraron diferencias notables entre un sexo y otro, lo que demuestra la utilidad e importancia del uso del CBCT en la estimación del sexo para un antropólogo y odontólogo forense.

Así mismo, Kazmi *et al*. ^28^ analizaron el volumen de la cavidad pulpar en caninos maxilares y mandibulares, que varía según la deposición de la dentina secundaria que la rodea, es decir, a mayor cantidad de dentina secundaria depuesta, menor será el volumen de la cavidad pulpar. Este factor podría considerarse un predictor para estimar la edad cronológica. Observaron, mediante TCHC, imágenes de 521 caninos maxilares izquierdos y 681 caninos mandibulares izquierdos recolectados de 368 mujeres y 349 hombres de entre 15 y 65 años. Encontraron que existe una mayor relación con la edad cronológica en los volúmenes de las cavidades pulpares de los caninos mandibulares que en los volúmenes de las cavidades pulpares de los caninos maxilares. Aunque no hallaron una relación lineal entre edad cronológica y volumen de cavidad pulpar de caninos mandibulares, existe cierta orientación para estimar edad cronológica.

Adicionalmente, gracias a sus ventajas en calidad de imagen, la TCHC es de mucha utilidad para la identificación de cadáveres en casos de desastres, ya que pueden utilizarse las imágenes producidas y contrastarse con fotografías y otras proyecciones radiográficas como la lateral de cráneo ^29^.

Así, se considera que la imagenología cumple un rol importante en la identificación, mediante la comparación de imágenes *pre* y *post mortem*, especialmente si se trata de casos de desastres. Además, si este hecho se complementa con elementos de tecnología moderna, puede hablarse entonces de la aplicación de herramientas computarizadas para investigar situaciones legales utilizando evidencia digital ^30^^,^^31^.

## LA RECONSTRUCCIÓN FACIAL FORENSE A PARTIR DE IMÁGENES

De esta manera, se puede conocer acerca de la reconstrucción facial forense. Esta técnica consiste en la estimación del modelo facial de un sujeto a partir de las estructuras craneales ^32^. Existen variados métodos para realizar la reconstrucción facial, métodos manuales que reproducen imágenes en 2 dimensiones o esculturas tridimensionales; en estos métodos se obtienen réplicas del cráneo y se le agregan marcas que indicarán los rasgos anatómicos que, posteriormente, definirán el grosor de los tejidos para ir reconstruyendo y esculpiendo el modelo facial estimado ^33^. Otros métodos, y los mayormente utilizados actualmente, son los que utilizan programas de *software*, pues tienen más ventajas que los manuales ya que presentan más herramientas para visualizar el cráneo y permiten estimar mejor el grosor tisular de la cara, evaluar más detalladamente el proceso de reconstrucción en incluso corregir errores ^34^.

En relación con lo mencionado, puede señalarse el trabajo de Meundi *et al*. ^35^, quienes buscaron proveer una base de datos del grosor tisular observable en una TCHC de una población del sur de India para posibles reconstrucciones faciales y evaluar diferencias raciales y de sexo, de haberlas. Ellos midieron el grosor tisular de 34 puntos craneométricos en 80 tomografías de personas entre 18 y 80 años, de una población del sur de India, y pudieron recolectar de forma exitosa una base de datos bastante amplia para facilitar la reconstrucción facial en caso de muerte o de posibles cirugías reconstructivas, destacando la importancia del uso de la TCHC en la reconstrucción facial forense.

En este marco, Kronseder *et al*. ^36^ reconstruyeron 150 ramas mandibulares de forma tridimensional. A partir de imágenes de TCHC, elaboraron el grosor del hueso, el alto y el ancho de la rama, así como el ángulo goníaco, para establecer parámetros digitales con relevancia clínica y forense. Lo que da importancia a su estudio es que puede servir como base para posteriores reconstrucciones faciales.

## DISCUSIÓN

Las estructuras óseas son un elemento esencial para la identificación en la odontología y la antropología forense, y la TCHC es la modalidad diagnóstica más utilizada actualmente por las características de las imágenes digitales que brinda. De hecho, algunos investigadores buscaron relacionar la odontología forense con la endodoncia, utilizando las imágenes producidas por la TCHC para evaluar el efecto de la temperatura en dientes tratados endodónticamente, como la hacen Patel *et al*. ^37^, quienes tomaron 40 premolares mandibulares tratados endodónticamente y los sometieron a temperaturas de 400 y 800 °C por 15 minutos (grupos de 20 dientes a cada temperatura), y realizaron tomografías a cada diente posteriormente. Se realizaron análisis odontológicos forenses macro y microscópicos a cada diente *pre* y *post mortem*, y se concluyó que todas las unidades dentarias sufren cambios macroscópicos y microscópicos a altas temperaturas, y si son analizados mediante la TCHC el estrés térmico produce cambios notables en la obturación tridimensional de los dientes tratados, por lo que resulta de utilidad en las ciencias forenses para la identificación de un cadáver calcinado.

Puede, además, señalarse el análisis de la mandíbula mediante la TCHC para estimar la edad o el sexo. La mandíbula es uno de los huesos craneales que, además de conservarse mejor después de la muerte, bien sea en caso de desastres, accidentes, calcinaciones u otros, tiene características anatómicas que permiten estimar la edad, el sexo o la incidencia racial ^38^^,^^39^.

En esta línea, Shaheen *et al*. ^40^ tomaron 10 imágenes de mandíbulas de tomografías computarizadas *pre mortem* y 10 imágenes con CBCT *post mortem* (muerte simulada) de los mismos individuos; en estas proyecciones se evaluó detalladamente la forma de la línea media mandibular de cada individuo. Las imágenes se utilizaron para contrastarlas entre sí y utilizarlas como medio de identificación. Aunque observaron diferencias significativas, el estudio mostró una efectividad del 100% para determinar la identidad de los sujetos, observando las características anatómicas de su línea media en las imágenes tomográficas *pre mortem* y contrastándolas con su TCHC *post mortem*.

Por su parte, Tassoker *et al*. ^41^ quisieron comparar el uso de la TCHC con la proyección panorámica para el análisis morfométrico de la mandíbula, tomaron 50 hombres y 71 mujeres divididos por sus grupos etarios en edades de 10 a 19, 20 a 29, 30 a 39, 40 a 49, 50 a 59 y 60 a 69 años, para un total de 121 sujetos de estudio, a quienes les fue realizado un análisis morfométrico de la mandíbula. Las mandíbulas masculinas presentaban caracteres anatómicos más prominentes que las femeninas en todos los grupos etarios y en ambas proyecciones, panorámica y TCHC; además, los valores de medidas mandibulares arrojados en las proyecciones panorámicas eran mayores que las observadas mediante una tomografía. Esto indica que la proyección panorámica presenta un mayor margen de error que la imágenes obtenidas mediante la TCHC, y también señala el valor de esta última modalidad diagnóstica como una herramienta para el odontólogo forense en la estimación de edad y sexo.

Complementando, Okkesim *et al*. ^42^ estudiaron los diferentes parámetros morfométricos mandibulares observables en una TCHC de 70 individuos (35 hombres y 35 mujeres), en edades promedio de 24 años, analizando las medidas de distintos puntos de la rama de la mandíbula, tanto del lado izquierdo como del derecho, en milímetros, mediante métodos especiales. Se destacaron variables morfométricas significantes entre ramas mandibulares masculinas y ramas mandibulares femeninas, es decir, mediante el análisis morfométrico de la rama de la mandíbula puede describirse de forma bastante clara el dimorfismo sexual, cuestión bastante importante para el antropólogo u odontólogo forense en la estimación del sexo.

Para describir otro de los usos de la TCHC en la odontología forense, puede mencionarse el estudio de Kulczyk *et al*. ^43^, quienes compararon la certeza de diferentes protocolos de reconstrucción tridimensional de molares y caninos mediante impresoras en 3D a partir de imágenes de la TCHC. Los resultados obtenidos en la reconstrucción de estas unidades dentarias con protocolos que utilizaban el escaneo óptico, a partir de imágenes de tomografías de alta resolución, fueron bastante buenos, ya que se observaron desviaciones bastante bajas del promedio de medida de estos dientes. Por el contrario, los modelos de reconstrucción obtenidos de protocolos que usaban como base una imagen de alta resolución de TCHC y una impresora tridimensional, los protocolos de escaneo óptico fueron significantemente más bajos. Por ello, para la reconstrucción de unidades dentarias, el TCHC y otras herramientas de última tecnología, como las impresoras en 3D, son de gran utilidad, e incluso esto podría servir como base para una posible reconstrucción facial, esencialmente si se encuentran casos de desastres masivos ^44^.

Tratando nuevamente la estimación de sexo, Farhadian *et al*. ^45^ estudiaron el dimorfismo sexual observando el proceso mastoideo y su posición en el cráneo en una población iraní mediante el uso de TCHC, observaron 190 tomografías de 105 mujeres y 85 hombres de edades entre 18 y 70 años, en las cuales midieron la distancia entre los puntos cefalométricos el porion y el mastoidale; la longitud, la altura y el ancho del proceso mastoideo; la distancia entre el mastoidal y la incisión mastoidea; la distancia intermastoidea (IMD); la distancia entre el punto más bajo del triángulo mastoideo y la superficie convexa más prominente del mastoideo (MF); la distancia entre el punto mastoideo convexo más prominente (IMSLD); y el ángulo de intersección dibujado desde el punto mastoideo derecho e izquierdo más prominente (MMCA).

De estas medidas, los investigadores encontraron una diferencia bastante significativa entre procesos mastoideos masculinos y femeninos en todas las variables, excepto la distancia entre el punto más bajo del triángulo mastoideo y la superficie convexa más prominente del mastoideo y la distancia entre el punto mastoideo convexo más prominente. A pesar de ello, la distancia intermastoidea y las demás variables muestran notables diferencias, por lo que es de utilidad para un odontólogo o antropólogo forense para estimar el sexo analizando el proceso mastoideo mediante la TCHC.

De la misma manera, Urooge *et al*. ^46^ basaron su estudio en el hecho de que, para la estimación de sexo, generalmente, los huesos que se encuentran están en estado de descomposición o incompletos, por lo tanto, utilizaron la TCHC para observar el seno maxilar y obtener información de este según sus características anatómicas y que sea más fácil establecer el sexo. Ellos midieron el tamaño y el volumen del seno maxilar, en todos los sentidos (ancho, alto, área y perímetro), de 100 pacientes, 50 hombres y 50 mujeres. Compararon las medidas obtenidas y fueron bastante relevantes, ya que se mostraron diferencias plausibles entre senos maxilares masculinos y femeninos en todos los aspectos medidos. En el caso de los senos maxilares femeninos, estos mostraron medidas mucho mayores que las medidas del seno masculino, especialmente en relación al ancho del seno izquierdo, por lo tanto, el ancho del seno maxilar observado mediante una TCHC es de gran utilidad para estimación del sexo en las ciencias forenses.

En suma, las estructuras óseas, como se mencionó, son claves para el odontólogo y antropólogo forense, no solo para la identificación, sino también para la estimación de la edad, en primer lugar, y luego del sexo. Así, las modalidades diagnósticas de la imagenología dentofacial facilitan el estudio de estructuras craneofaciales, como el seno maxilar, la mandíbula y otros ^47^^,^^48^.

## CONCLUSIONES

En la odontología forense, para identificar, estimar la edad o el sexo, y la proyección panorámica, son bastante útiles la radiografía lateral de cráneo, la radiografía posteroanterior de cráneo y la tomografía computarizada de haz cónico.

Una radiografía panorámica, la posteroanterior y lateral de cráneo, permiten el estudio y el análisis de la mandíbula y de las unidades dentales para identificar un cadáver; además, sirve para estimar la edad de un sujeto vivo cuya edad se desconozca o la de un cadáver. También, si se realizan estudios profundos de la mandíbula observada en esta proyección, puede estimarse el sexo del sujeto, además de ser útiles para una posible reconstrucción facial.

La tomografía computarizada de haz cónico resulta de gran utilidad y mucho uso en la actualidad desde un punto de vista forense, ya que brinda imágenes digitales en planos que permiten tener una visión tridimensional al cráneo, esto detalla de forma más nítida esta estructura anatómica y permite o facilita, no solo las labores de identificación, la estimación de edad o sexo, sino que también puede elaborarse una reconstrucción facial forense detallada a partir de las imágenes obtenidas en esta modalidad diagnóstica. 

## Fuente de financiamiento:

Autofinanciado
